# The Removal of the Tooth Pulp by the Use of Cocaine

**Published:** 1893-04

**Authors:** Edward C. Briggs

**Affiliations:** Boston, Mass.


### The Removal of the Tooth Pulp by the Use of Cocaine.

BY EDWARD C. BRIGGS, M.D., OF BOSTON, MASS.

Read in the Section of Oral and Dental Surgery, at the Forty-third Annual Meeting of the American Medical Association, held in Detroit, Mich., June, 1892.

In October, 1890, I read a paper on this subject before a society in Boston and recounted many successful operations done since the preceding May. Since then I and some of my neighboring associates have been so successful that I feel constrained to bring the matter before this Association for a wider field in which to sow such good seed. In almost all cases of exposed pulps the pulp can be paralyzed and removed with no pain or discomfort to the patient. Cases which would seem most antagonistic to any radical treatment yield most gracefully to this.

Pulps of known irritability which would resist arsenious acid are satisfactorily disposed of by this method. Teeth are not "devitalized" by this treatment, as is so often the case with strong caustics, but are simply made "pulpless"—retaining their color and vital connection with the jaw.

The immediate root filling which is a part of the operation seals the tooth before germs can get in, and there is no abscess to be feared.

To operate it is necessary to have: 1. A tightly packed, smoothly running hypodermic syringe, with a flexible, blunt point. This latter condition I obtain by putting the point of a Dunn syringe into a hypodermic syringe.

2. A *freshly* prepared 20 per cent. solution of hydrochlorate of cocaine. (I have not yet had the opportunity to experiment with so-called milk of cocaine, which is suggested as being better.)

Having an exposure of the pulp (the smaller the better,) first apply on cotton some of the cocaine solution. This in a few moments makes the exposed part approachable, and if the opening through the dentine into the pulp is not as large as the end of the syringe point the syringe can be immediately placed over the point of exposure.

Having adjusted the syringe point, the piston is then to be firmly and steadily pressed down slowly at first and with increasing pressure, finally to be pressed down with considerable force, carrying the solution up and around the pulp to its extreme end. At the first impact of the liquid against the pulp the patient sometimes feels a shock—not worse, however, than one gives the patient when feeling with a probe to see if previous application of arsenious acid has done its work. If now the syringe point has been properly directed and fits over the exposure or into the opening in dentine, so as to allow but little back flow, the operator can immediately open freely into the pulp chamber with a bur, remove the pulp from the canals with barbed broaches; or even when possible, enlarge the canals and remove the pulp at same time with a Gates-Glidden drill.

The advantage of this latter method lies in the fact that after the pulp has been entirely removed by a broach, if there is any delay in putting in the filling the inner walls of root or roots become sensitive again and it is as impossible to pass a probe into them as when the pulp was *in situ*.

A case or two by way of illustration:

Miss L. had been in Philadelphia during the winter and having gone away without the usual autumn examination a capped pulp had come to grief and an eminent dentist in Philadelphia had applied, presumably, arsenious acid. The patient returned to Boston with cotton dressing in the tooth and reported to me the day before departing for Europe, wishing to have the tooth filled. She said the tooth was "dead," but when I passed a probe into the distal root, I found it very much alive. It took but a moment, however, to pass a small syringe point down into the root, inject the cocaine rather forcibly, and then with barbed broach remove quite a sizable piece of root pulp. The roots were then washed with antiseptics, dried, and filled with gutta percha.

This case is typical of a large class, and we all have or have had teeth with "life in the end of the roots" which have baffled our efforts to completely remove, and which therefore we have had to keep dressed and temporarily filled for months and even years waiting for complete death. As a fellow practitioner remarked to me the other day, the operation was worth everything for just those cases alone.

Mrs. R. is a patient of my brother's. He was called to see her for the first time in her sick room where she had been a nervous invalid for over a year. Two or three teeth presented exposed pulps. My brother thought to disturb her least by making application of arsenious acid. It was with the too often result —inflammation, pain, and no destruction of pulp. He then tried the method described here, and removed the pulps of two teeth without the slightest shock or pain to the patient. This too under the most disadvantageous circumstances at the residence of the patient. I have the records of fifty or more cases almost all of which can be classed as perfectly successful. Where failure has been the result it has been due to defect in carrying out the details of the operation as described; failure to get syringe point directly over exposure; failure to have syringe in perfect working order; failure to use at least a 20 per cent. solution; and last but not least, neglect to have a fresh solution of the drug.

The operation must be done carefully, methodically—exactly as we specialists have to do our work. When so done it will prove a success and a great boon to the profession. It is hardly necessary to add that proper care must be taken to prevent the patient swallowing any of the drug, using, as is necessary, so strong a solution.

---

THE laws of Michigan forbid any railroad within its borders to employ other than total abstainers from alcoholics as engineers, train dispatchers, firemen, brakemen, or other railroad servants. The penalty is said to be $500 for each offense. Yet many of the passenger boats in said State have open bars on board for the patronage of all.—*American Lancet.*

